# Total hip arthroplasty and perioperative management of a patient with hip osteonecrosis secondary to hypopituitarism due to Sheehan syndrome: a case report and literature review

**DOI:** 10.3389/fsurg.2026.1715057

**Published:** 2026-02-04

**Authors:** Biao Ma, Jun Li, Tao Ma, He Shang, Xueqi Liu, Tianxiang Yang, Jinpeng Liang, Yaxing Ma, Ruoyu Wang, Hui Ma, Jiao Liu, Desheng Chen

**Affiliations:** 1 Third Clinical Medical College of Ningxia Medical University, Yinchuan, China; 2 Department of Joint Surgery, Ningxia Hui Autonomous Region People’s Hospital (Affiliated Hospital of Ningxia Medical University), Yinchuan, China; 3 Laboratory Medicine of Ningxia Medical University, Yinchuan, China

**Keywords:** multidisciplinary team, osteonecrosis of the femoral head, perioperative management, Sheehan's syndrome, total hip arthroplasty

## Abstract

**Background:**

Sheehan's syndrome, a rare disorder resulting from postpartum hemorrhage-induced necrosis of the anterior pituitary gland, necessitates long-term hormone replacement therapy with glucocorticoids. This predisposes patients to severe complications, including rapid-progression osteonecrosis of the femoral head (ONFH), which carries a high disability rate.

**Methods:**

We report a 63-year-old female with Sheehan's syndrome diagnosed 30 years ago, managed with sustained prednisone and levothyroxine. She presented with 10 years of bilateral hip pain and imaging-confirmed bilateral ONFH at ARCO stage IV. A multidisciplinary team (MDT) approach was implemented: endocrinology optimized preoperative hormone regimens, orthopedics planned total hip arthroplasty (THA) based on bone density assessments, and anesthesiology confirmed tolerability for intraspinal anesthesia. After stabilizing physiological parameters, left THA was performed.

**Results:**

Intraoperative hydrocortisone infusion maintained hormonal homeostasis. The surgery proceeded uneventfully; however, an allergic reaction occurred during blood transfusion, which was promptly controlled. Postoperative MDT-coordinated care enabled ambulation with a walker within one week, with unrestricted left hip mobility and no complications (e.g., infection, prosthesis loosening).

**Conclusion:**

THA for glucocorticoid-induced ONFH in Sheehan's syndrome entails challenges such as perioperative hormonal instability, elevated infection risk, and impaired bone healing. The MDT approach ensures comprehensive risk mitigation, facilitating surgical success and patient safety. Long-term follow-up for hormone levels, bone density, and prosthesis status is warranted.

## 1 Introduction

Sheehan's syndrome is an endocrine disorder characterized by ischemic necrosis of the anterior pituitary gland, typically resulting from postpartum hemorrhage and prolonged shock. This condition disrupts the regulation of multiple endocrine glands, leading to persistent deficiencies in hormones such as glucocorticoids, thyroid hormones, and sex hormones. Consequently, patients require lifelong exogenous hormone replacement therapy, including glucocorticoids for adrenal insufficiency. However, long-term glucocorticoid administration can precipitate severe complications, including osteonecrosisof the femoral head (ONFH), with increased risk in patients with concurrent hypothyroidism. ONFH is a debilitating hip disorder defined by impaired blood supply to the femoral head, resulting in ischemic necrosis of bone cells and marrow, trabecular bone loss, and subsequent femoral head collapse (1). The precarious vascular anatomy of the femoral head, with limited collateral circulation, contributes to rapid clinical progression from insidious hip pain to impaired mobility and joint dysfunction (2).Primary risk factors for ONFH include prolonged or high-dose corticosteroid therapy, excessive alcohol intake, and other contributors such as hypercholesterolemia or inflammatory diseases (3). The pathophysiology involves steroid-induced damage to vascular endothelial cells, formation of microthrombi, and abnormal bone marrow adipogenesis; hypertrophied adipocytes compress blood vessels and exacerbate ischemia, while elevated reactive oxygen species (ROS) and disrupted osteoblast-osteoclast homeostasis accelerate bone destruction (4–6). As ONFH advances to femoral head collapse or secondary osteoarthritis, total hip arthroplasty (THA) serves as the definitive end-stage treatment (7). In Sheehan's syndrome patients undergoing THA, perioperative management necessitates stringent hormone optimization, vigilant thyroid function monitoring, and early rehabilitation with restricted weight-bearing to reduce complication risks.By searching the PubMed and Embase databases from 2014 to 2024, using the keywords “Sheehan syndrome” AND “total hip arthroplasty” AND “osteonecrosis of the femoral head”, we excluded reviews, studies with fewer than 3 cases, and non-English literature. Finally, 16 relevant case reports or small-sample studies were included to analyze the perioperative management characteristics of patients with Sheehan syndrome undergoing total hip replacement. Clinically, due to the irreversible nature of anterior pituitary necrosis in patients with Sheehan syndrome, long-term hormone replacement therapy (HRT) remains essential for maintaining basic physiological functions. However, glucocorticoid-related osteonecrosis of the femoral head (ONFH) represents a significant and well-recognized complication of this therapeutic regimen (5, 8). The risk of developing ONFH correlates strongly with glucocorticoid dosage, treatment duration (notably increasing with durations exceeding 5 years), and individual patient factors, including concurrent conditions such as hypothyroidism and osteoporosis. Risk stratification for high-risk patients is critical, guiding the implementation of enhanced surveillance protocols to ensure diligent risk management. Clinical practice adheres to a “prevention first and early intervention” principle (7). This involves initiating HRT with individualized treatment plans, aiming for the minimal effective glucocorticoid dose necessary for physiological replacement.

Regular monitoring is paramount, including hip joint magnetic resonance imaging (MRI) and bone mineral density (BMD) assessments. Hip pain or discomfort warrants prompt investigation for potential ONFH (9). For early-stage ONFH (ARCO stages I-II), conservative interventions are employed to slow disease progression. When the disease advances to ARCO stage IIIb or greater, total hip arthroplasty (THA) becomes the primary therapeutic approach. Management adopts a multidisciplinary team (MDT) approach. Endocrinology leads hormone level optimization and management of associated comorbidities. Orthopedics oversees ONFH staging assessment, surgical intervention when indicated, and post-operative monitoring for complications such as prosthetic dislocation or loosening (7, 9). Radiology provides accurate staging through MRI and computed tomography (CT) (9), while Rehabilitation Medicine designs tailored post-operative recovery programs.

Prognostically, secondary ONFH in Sheehan syndrome, managed with early intervention, is often controllable, effectively preserving hip joint function (6). Patients requiring THA typically regain ambulatory capacity following structured rehabilitation. Lifelong follow-up is mandatory. Keyfollow-up components include assessing the efficacy of HRT to prevent adrenal crisis, evaluating the prosthetic joint via periodic hip radiographs, and vigilance for potential ONFH development in the contralateral hip joint.

## 2 Case presentation

A 63-year-old woman was admitted to our hospital with severe bilateral hip pain that had progressively worsened over the preceding 20 days, resulting in an inability to walk independently and significant impairment in daily activities. The patient reported a 10-year history of bilateral hip pain, during which conservative treatments, including physical therapy, failed to provide sustained symptom relief.

Her medical history was notable for severe postpartum hemorrhage 30 years earlier during her third pregnancy, after which she developed Sheehan syndrome. Subsequent evaluation confirmed hypopituitarism and secondary hypothyroidism, and she was prescribed long-term glucocorticoid and levothyroxine replacement therapy. The patient reported poor adherence to regular hormone monitoring and dose adjustment over the past two decades. She had no history of hypertension, diabetes mellitus, gallbladder disease, pancreatitis, or chronic infectious diseases. She was treated for brucellosis in 2023 with complete clinical resolution (Table 1).

**Table 1:** Timeline of the patient's clinical course with interpretive commentary.

Time node	Important events	Medical history and treatment process	Explanation of interpretation
In 1986 (at the age of 26)	During the third pregnancy at home, the placenta failed to separate properly, resulting in severe bleeding.	At Guoyuan City People's Hospital, the placenta was manually removed and blood transfusions were administered twice. After one month of hospitalization, the patient's condition stabilized and was discharged.	This severe postpartum hemorrhage led to necrosis of the anterior pituitary gland, which was the fundamental cause for the subsequent occurrence of Sheehan's syndrome and long-term hormone deficiency.
2002 (40 years old)	Menopause		
2003 (41 years old)	Due to fatigue, excessive sleepiness, loss of appetite, and even coma	At Zhongning County Traditional Chinese Medicine Hospital, considering thyroid disorders, blood samples were sent to the Affiliated Hospital of Ningxia Medical University for examination. The results indicated hypothyroidism and hypopituitarism. Prednisone and levothyroxine tablets were prescribed for control (the specific dosages are unknown).	After this treatment, the patient took prednisone and levothyroxine tablets (the specific dosages are unknown), and the hormone levels were not monitored subsequently.
2014 (50 years old)	Due to pain in both hip joints	At our Xixia Hospital District, a thorough examination confirmed bilateral femoral head necrosis. Due to hypopituitarism and hypothyroidism, the hormone levels were low. After the anesthesiologist's assessment, surgery was not feasible, so the patient was discharged for conservative treatment.	At this point, the patient's hormone levels were not properly regulated (insufficient cortisol + hypothyroidism), which increased the surgical risk. Therefore, conservative treatment was given instead of total hip replacement surgery.
2023 (61 years old)	Due to brucellosis	After successful treatment and discharge from the Fourth People's Hospital of Ningxia Hui Autonomous Region	
20 days ago (63 years old)	Due to severe pain in both hip joints, she is unable to walk.	In our hospital's emergency department, after completing all the necessary examinations and tests, we admitted the patient to the hospital.	After assessment by the multidisciplinary team, all relevant examinations and tests have been completed. The left total hip replacement surgery is scheduled to be performed in 1 day.

**Figure 1:**
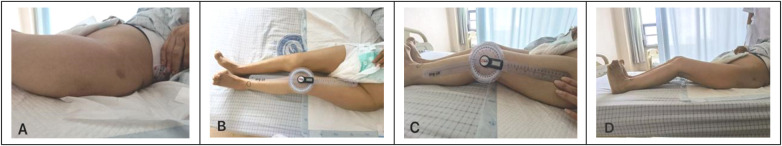
**(A)** Represents the local position of the left hip joint; **(B)** is the frontal view of the left hip joint; **(C)** is the side view (proximal) of the left hip joint; **(D)** is the side view (distal) of the left hip joint.

On physical examination, there was no visible swelling of either hip (Figure 1). Palpation elicited tenderness in the bilateral inguinal regions and pain on axial loading of both lower limbs. Range of motion of both hips was markedly restricted and associated with pain, particularly during internal and external rotation. The left hip showed flexion of approximately 60°, internal rotation of 5°, external rotation of 10°, abduction of 20°, and adduction of 10°. The right hip demonstrated flexion of approximately 70°, internal rotation of 15°, external rotation of 20°, abduction of 30°, and adduction of 20°. The FABER test was positive bilaterally. Muscle strength in the lower extremities was graded as 5/5 according to the Medical Research Council scale.

**Figure 2:**
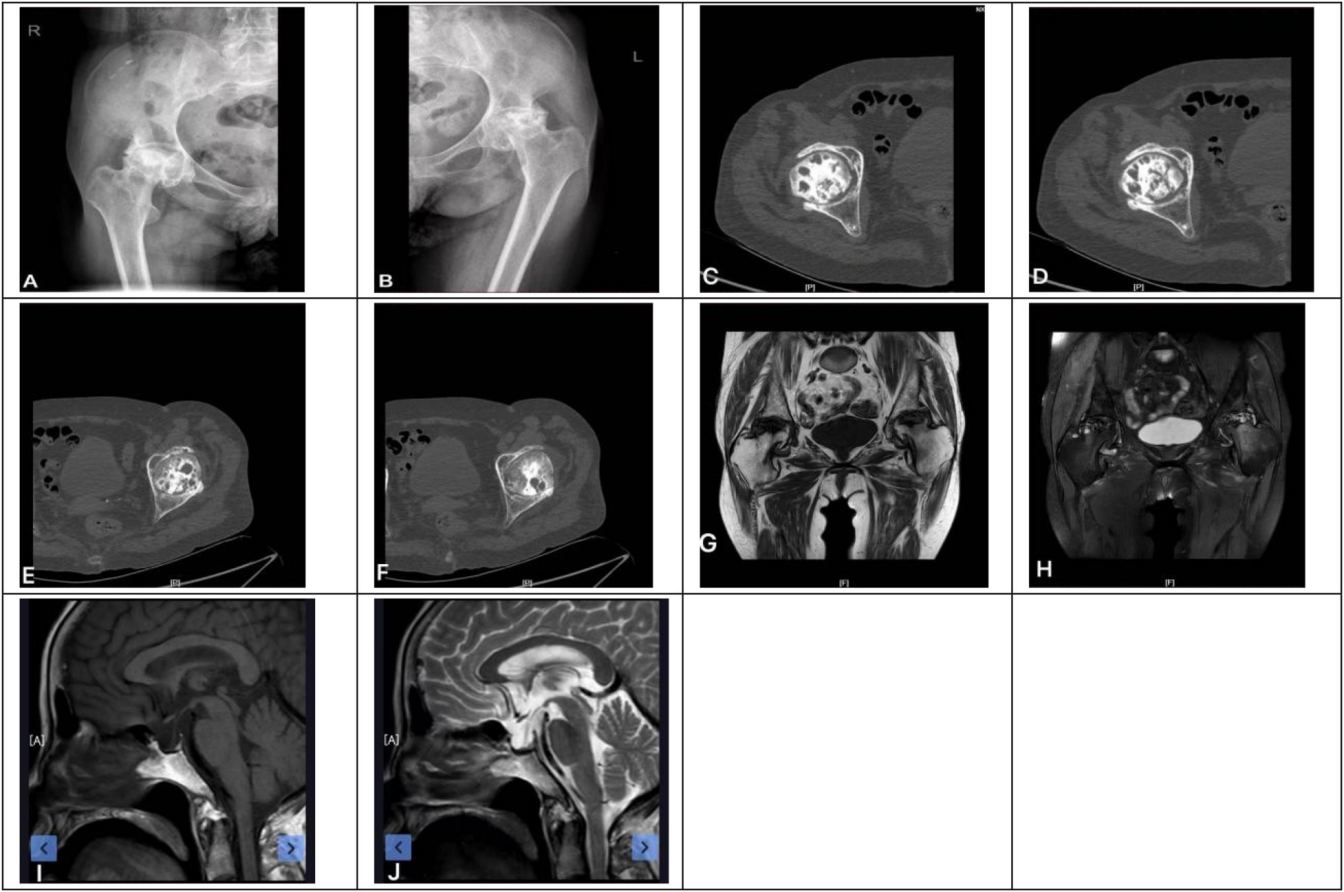
Preoperative imaging data. **(A)** Right hip anteroposterior x-ray (no obvious femoral head collapse); **(B)** Left hip anteroposterior x-ray (arrow indicates femoral head collapse and joint space narrowing); **(C,D)** right hip CT (subchondral cystic lesions, arrow); **(E,F)** left hip CT (acetabular sclerosis, arrow); **(G)** left hip T1-weighted MRI (patchy hypointensity in femoral head, arrow); **(H)** left hip T2-weighted MRI (hyperintensity in femoral head, arrow, consistent with edema); **(I,J)** head T1/T2-weighted MRI (empty sella, arrow).

Radiographic and imaging evaluations demonstrated advanced bilateral osteonecrosis of the femoral head (Figure 2). Plain radiographs and computed tomography revealed irregular femoral head morphology with collapse, subchondral cystic changes, sclerosis, and joint space narrowing. Magnetic resonance imaging showed patchy hypointense signals on T1-weighted images and hyperintense signals on T2-weighted fat-suppressed sequences in the femoral heads and necks, consistent with extensive osteonecrosis. The lesions involved the acetabulum, accompanied by secondary hip osteoarthritis. According to established staging criteria, the condition was classified as bilateral ONFH at stage IV. Brain magnetic resonance imaging revealed an enlarged sella turcica with flattening of the pituitary gland, consistent with an empty sella.

Bone mineral density assessment using dual-energy x-ray absorptiometry demonstrated osteoporosis in the lumbar spine (L1–L3) and osteopenia at L4, while bone mineral density at the left femoral neck remained within the normal reference range.

Laboratory investigations showed markedly reduced adrenocorticotropic hormone and cortisol levels, consistent with adrenal insufficiency. Thyroid function testing revealed reduced free triiodothyronine and triiodothyronine levels with suppressed thyroid-stimulating hormone, while free thyroxine and thyroxine levels were within normal limits, indicating secondary hypothyroidism (Table 2). Additional abnormalities included hyponatremia, hypochloremia, hypoalbuminemia, and elevated liver enzyme levels. Sex hormone analysis demonstrated reduced testosterone levels (Table 3).

**Table 2:** Serum adrenocorticotropic hormone and cortisol determination.

Name	Result	Organization	Reference range
Adrenocorticotropic hormone (ACTH)	2.487↓ (8AM Serum)	pg/mL	7.2–63.4
Adrenocorticotropic hormone (ACTH)	<1.000↓ (4PM Serum)	pg/mL	7.2–63.4
Cortisol (CORT)	11.50 (8AM Serum)	nmol/L	8AM Serum: 166—507; 4PM Serum: 73.8–291
Cortisol (CORT)	24.40 (4PM Serum)	nmol/L	8AM Serum: 166–507; 4PM Serum: 73.8–291
Free triiodothyronine (FT3)	1.84↓	pmol/L	3.1–6.8
Free thyroxine (FT4)	21.6	pmol/L	11.9–21.6
Triiodothyronine (T3)	1.07↓	nmol/L	1.3–3.1
Thyroxine (T4)	140	nmol/L	66–181
Thyroid stimulating hormone (TSH)	0.01↓	uIU/mL	0.27–4.2

**Table 3:** Liver function and electrolyte tests and sex hormone evaluations.

Name	Result	Organization	Reference range
Alanine aminotransferase (ALT)	89.9↑	U/L	7–40
Aspartate aminotransferase (AST)	43.9↑	U/L	13–35
Total protein (TP)	58.8↓	g/L	65–85
Albumin (ALB)	30.1↓	g/L	40–55
Globulin (GLB)	28.7	g/L	20–40
Albumin-to-globulin ratio (A/G)	1↓		1.2–2.4
Alkaline phosphatase (ALP)	436.6↑	U/L	50–135
Glutamyl transpeptidase (GGT)	142.1↑	U/L	7–45
Sodium (NA)	128↓	mmol/L	137–147
Chlorine (CL)	91↓	mmol/L	99–110
Estriol (E2)	80.7	pmol/L	Man: 41.4–159; Woman: Follicular phase: 114–332, Ovulation period: 222–1959, Luteal phase: 222–854, Menopause: 0–505
Follicle-stimulating hormone (FSH)	4.5	IU/L	Man: 1.5–12.4; Woman: Follicular phase: 3.5–12.5, Ovulation period: 4.7–21.5, Luteal phase: 1.7–7.7, Menopause: 25.8–134.8
Luteinizing hormone (LH)	2.09	IU/L	Man: 1.7–8.6; Woman: Follicular phase: 2.4–12.6, Ovulation period: 14.0–95.6, Luteal phase: 1.0–11.4, Menopause: 7.7–58.5
Progesterone (PROG)	<0.16	nmol/L	Man: 0–0.474; Woman: Follicular phase: 0–0.616, Ovulation period: 0.175–13.2, Luteal phase: 13.1–46.3, Menopause: 0–0.401, Early pregnancy35–141, Second trimester of pregnancy80.8–265, The third trimester of pregnancy187–679
Prolactin (PRL)	202	uIU/mL	102–496
Testosterone (TESTO)	<0.09↓	nmol/L	0.101–1.42

Given the advanced stage of osteonecrosis and severe functional limitation, the patient was evaluated by a multidisciplinary team comprising endocrinology, orthopedics, anesthesiology, nutrition, and rehabilitation specialists. After comprehensive preoperative assessment and optimization, left total hip arthroplasty was planned and performed.

## 3 Treatment

Preoperative Management: We designed a stepwise workflow covering three core phases: “preoperative optimization (7–3 days preoperatively), intraoperative collaboration (surgery day), and postoperative follow-up (1–90 days postoperatively).”

Preoperative Optimization Phase: In the first MDT meeting held 7 days preoperatively, the Department of Endocrinology proposed an initial hormone adjustment plan (prednisone 5 mg in the morning/2.5 mg in the evening) based on baseline hormone levels [e.g., cortisol 58 nmol/L, free triiodothyronine (FT3) 1.84 pmol/L]. The Department of Orthopedics reported the ARCO Stage IV classification of osteonecrosis of the femoral head (ONFH) and the surgical plan for total hip arthroplasty (THA) via the posterolateral approach. The Department of Nutrition recommended 370 kcal/day of oral nutritional supplements for hypoalbuminemia (albumin 30.1 g/L). It was finally confirmed that further hormone optimization and nutritional support were needed, and a second meeting was scheduled for 5 days preoperatively. In the second meeting held 5 days preoperatively, the Department of Endocrinology reported the adjusted hormone levels (cortisol 82 nmol/L) and clarified the stress protocol of 100 mg intravenous hydrocortisone during surgery. After assessing the patient's ASA Class III risk, the Department of Anesthesiology proposed a spinal anesthesia plan (to avoid endocrine stress induced by general anesthesia). The orthopedic department discussed the selection of the non-cemented Aikang ACT socket and CL handle prosthesis. Finally, the anesthesia plan and prosthesis type were determined, and the patient's pelvic inclination angle (approximately 5°) was evaluated through CT. The prosthesis installation angle was adjusted according to the literature of Ramadanov N to avoid postoperative hip joint impact (10). At the same time, the preoperative preparation list such as correcting hyponatremia with hypertonic saline was confirmed. In the third meeting three days before the surgery, the endocrinology department verified the final stable state of hormones (cortisol 95 nmol/L, thyroid stimulating hormone 0.32 uIU/mL). The Department of Anesthesiology confirmed the feasibility of spinal anesthesia and prepared emergency drugs (e.g., epinephrine, dexamethasone). All departments jointly signed the final perioperative management plan, approved the patient's eligibility for surgery, and archived the meeting minutes in the electronic medical record.

Intraoperative Collaboration Phase: The Department of Anesthesiology monitored invasive arterial blood pressure and core body temperature, and administered stress-dose hydrocortisone at the time of skin incision. The Department of Endocrinology stood by to manage hormonal crises (e.g., adrenal insufficiency). The orthopedic department will promptly relay the findings from the operation (such as femoral head collapse) to the team, and the team will jointly address adverse events (such as plasma infusion allergy) to ensure the hormonal balance during the operation.

Postoperative Follow-up Phase: Within 1–7 days postoperatively, the Department of Endocrinology guided the tapering of hydrocortisone from 80 mg to 40 mg orally. The Department of Orthopedics monitored incision healing and prosthesis position via x-ray on day 4 postoperatively. The Department of Rehabilitation designed a walker-assisted ambulation training program (10 min/day starting from day 3 postoperatively). The team assessed the Visual Analogue Scale (VAS) pain score and Harris Hip Score through weekly MDT rounds and adjusted the rehabilitation intensity accordingly. Between 30 and 90 days postoperatively, the Department of Endocrinology rechecked long-term hormone levels (cortisol 120 nmol/L). The Department of Orthopedics evaluated prosthesis stability via CT on day 90 postoperatively. The Department of Nutrition confirmed the normalization of albumin (38.5 g/L). A long-term follow-up plan (“hormone monitoring every 3 months and bone mineral density assessment annually”) was finally established.

All MDT meeting minutes (including scanned copies of signatures and presentation PPTs) are archived in the electronic medical record system of Ningxia Hui Autonomous Region People's Hospital. The archive numbers are: NMUH-2024-THA-MDT-01 (7 days before the surgery), NMUH-2024-THA-MDT-02 (5 days before the surgery), NMUH-2024-THA-MDT-03 (3 days before the surgery). They can be requested for retrieval and verification through the hospital's ethics committee.

Preoperative period: To mitigate surgical risks, enhance procedural safety, and facilitate postoperative recovery, a preoperative multidisciplinary team (MDT) assessment was conducted, involving specialists from endocrinology, anesthesiology, and nutrition departments. This team collaboratively formulated an individualized preoperative preparation plan tailored to the patient's complex endocrine and metabolic condition.

The endocrinology evaluation utilized post-admission endocrine function test results to refine the hormone therapy regimen. Specifically, prednisone was adjusted to 5 mg orally each morning and 2.5 mg each evening, with levothyroxine maintained at 25 μg daily. Daily monitoring of serum cortisol and thyroid function (FT3, FT4, TSH) was implemented to stabilize hormonal parameters within defined thresholds for surgical tolerance, thereby minimizing risks associated with hormonal fluctuations. For stress response prophylaxis, considering the potential for surgical trauma to induce adrenal crisis, intravenous hydrocortisone 100 mg was administered intraoperatively; postoperative tapering of this therapy was guided by vital signs and hormonal assays to avert stress-related disorders. Concurrently, hyponatremia and hypochloremia were addressed through intravenous infusion of a hypertonic saline solution (10% sodium chloride at 3 g in 250 mL of 0.9% saline), with serial electrolyte assessments to monitor response.

Anesthesiology assessment, integrating endocrine stability and systemic factors, advocated for spinal anesthesia over general anesthesia to reduce endocrine system stimulation and optimize anesthetic efficacy. Preoperative preparations included prophylactic blood product management, with crossmatched red blood cells (2 units) and fresh frozen plasma (200 mL) reserved for hemorrhage control, alongside emergency medications such as stress-dose hydrocortisone and anti-allergic agents (e.g., epinephrine and dexamethasone) for managing adverse reactions or transfusion-related complications.

**Figure 3:**
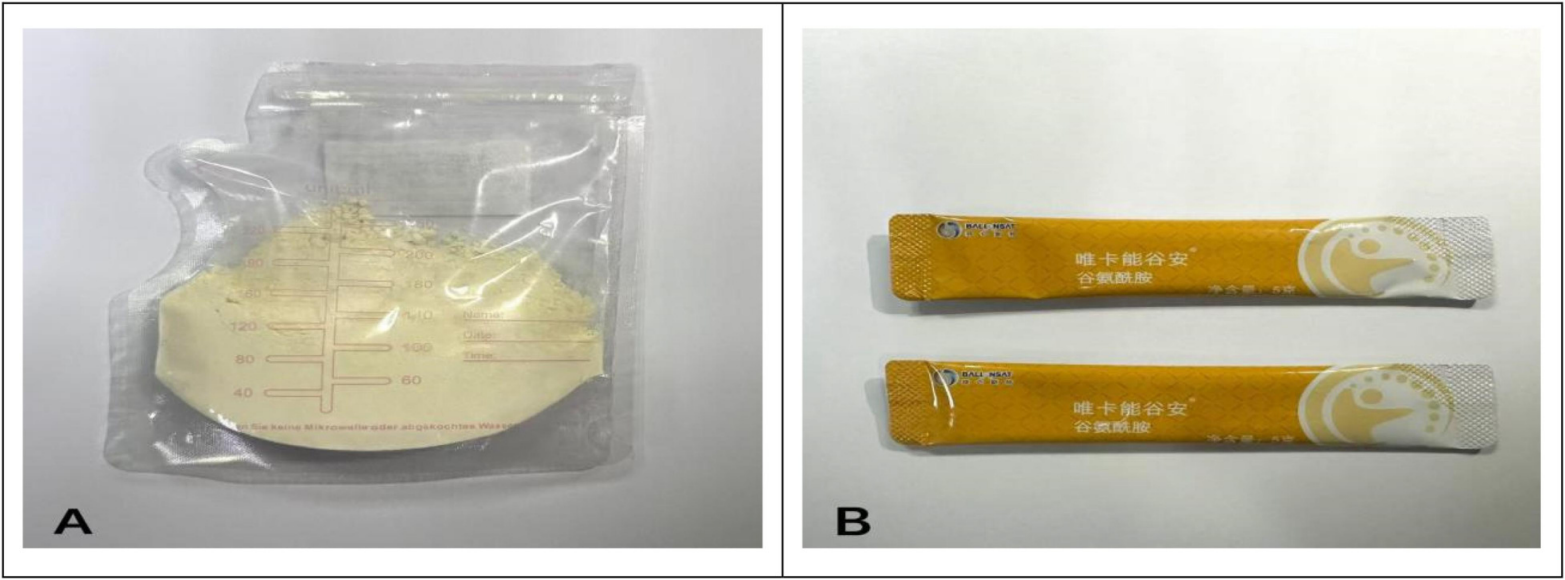
**(A)** represents the nutritional supplement; **(B)** represents glutamine.

Nutritional status appraisal employed body mass index (BMI, 21.5 kg/m²), Nutritional Risk Screening (NRS2002, score 1), Subjective Global Assessment (SGA, Grade A), and serum albumin levels (30.1 g/L; normal 35–50 g/L), revealing hypoalbuminemia that could impair surgical resilience. Preoperative nutritional support comprised perioperative dietary counseling, administration of a complete oral nutritional supplement (providing approximately 370 kcal, with 21 g protein, 10 g fat, and 46 g carbohydrates), and glutamine supplementation (10 g dissolved in 50 mL warm water) prior to surgery to bolster wound healing and recovery (Figure 3).

In this case, the MDT team approach, which is a comprehensive MDT method, demonstrated individualized preoperative optimization. Our outcome (0% complication rate) appears favorable compared to the 33% rate reported in some small case series of THA in Sheehan's syndrome patients, though direct comparison is limited by sample size.

Intraoperative period: The anesthesiologist inserted a 14G double-lumen catheter into the right internal jugular vein to monitor central venous pressure, and placed a 20G catheter into the left radial artery to continuously monitor invasive arterial blood pressure. Thirty minutes before anesthesia induction, ultrasound-guided bilateral femoral nerve blocks were performed (20 mL of 0.375% ropivacaine per side). Anesthesia was administered via spinal anesthesia at the L3-L4 interspace (2.5 mL of 0.75% bupivacaine injected, with the anesthetic level controlled below T10). Throughout the operation, a warm air blanket was used for surface heating (set at 38 °C) to maintain the patient's core body temperature between 36.0–37.0 °C.

With satisfactory anesthesia (BIS value 40–60), the patient was positioned in the lateral decubitus position (affected side up). The surgical field was disinfected with povidone-iodine solution (ranging 20 cm above the anterior superior iliac spine, down to the knee, and medially to the perineum) and draped with sterile towels. A posterolateral longitudinal incision (approximately 15 cm in length, starting 5 cm below the posterior superior iliac spine and extending 3 cm below the greater trochanter) was made on the affected hip. The skin and subcutaneous tissue were incised sequentially (with hemostasis achieved via electrocoagulation), followed by incision of the tensor fasciae latae and gluteus maximus fascia. The interval between the gluteus maximus and gluteus medius was bluntly dissected posterior to the greater trochanter, and the sciatic nerve was retracted posteriorly with a moist gauze (with continuous monitoring of sciatic nerve motor potentials to ensure neural integrity). After retracting the gluteus medius, the external rotators (piriformis, superior gemellus, obturator internus, and inferior gemellus) were exposed, clamped at their insertion on the intertrochanteric fossa, transected with an electrocautery (and marked for subsequent suture), revealing the lesser trochanter and posterior hip joint capsule. The posterior capsule was incised in a T-shape at its insertion (preserving a 1 cm margin for repair), and intra-articular effusion and adhesions were cleared.

The hip joint was flexed to 90° and internally rotated to 30° to dislocate the femoral head, which showed obvious osteonecrosis with deformation and collapse; the posterior acetabulum exhibited osteophyte formation and sclerosis. A femoral neck osteotomy was performed 1.5 cm above the lesser trochanter using an oscillating saw (osteotomy plane at 45° to the femoral neck axis). After removing the femoral head, the intra-acetabular synovium, ligamentum teres, and labrum were excised. The acetabulum was sequentially reamed with 44 mm–50 mm reamers until complete removal of cartilage and uniform subchondral bone bleeding was achieved. A 50 mm trial acetabular component (Aikang ACT series, cementless) was inserted with 40° abduction and 15° anteversion; fluoroscopy confirmed proper fit with >90% bone coverage. The trial component was removed, the acetabulum was irrigated, and a 50 mm Aikang ACT-50 cementless acetabular cup was implanted, secured with two 4 mm × 25 mm cancellous screws at the anterosuperior and posterosuperior acetabular quadrants, followed by insertion of a 32 mm inner diameter polyethylene liner.

A 1st femoral canal drill was used to open the medullary cavity 1 cm medial to the apex of the greater trochanter, followed by sequential reaming with 2nd and 3rd reamers. A 3rd trial femoral stem (Aikang CL series, cementless) and a trial ceramic head (size M, 32 mm outer diameter) were inserted. Joint reduction was performed, and stability was verified (no dislocation with 30° internal rotation or 40° external rotation, no loosening on longitudinal percussion) with <5 mm lower limb length discrepancy. The trial components were removed, the femoral canal was irrigated, and a 3rd Aikang CL-3# cementless femoral stem and a ceramic head (size M, 32 mm outer diameter) were implanted. Repeat reduction confirmed proper component fit, joint stability, and near-equal lower limb lengths.

Adequate hemostasis was achieved (using bipolar electrocoagulation for the transected external rotators and periacetabular vessels), and the incision was irrigated repeatedly before placing a 14F silicone drain. The external rotators were sutured *in situ* (with 2-0 absorbable sutures) and the gluteus medius insertion was repaired (with 1-0 non-absorbable sutures). The fascia lata, subcutaneous tissue, and skin were closed sequentially (with 4-0 absorbable sutures for intradermal skin closure). Tranexamic acid (2 g) was injected into the joint space, and the incision was covered with sterile dressings and compressed with an elastic bandage.

The total operative time was 110 min, with an estimated blood loss of 400 mL. The patient received 400 mL of leukocyte-depleted homologous red blood cells and 400 mL of fresh frozen plasma. Approximately 100 mL into plasma transfusion, the patient developed a World Allergy Organization (WAO) Grade II anaphylactic reaction (generalized pruritus, diffuse erythema, urticaria, arterial blood pressure dropping to 75/45 mmHg, heart rate 115 bpm). Plasma transfusion was immediately stopped; 1 g of 10% calcium gluconate and 10 mg of dexamethasone were rapidly administered intravenously, and epinephrine was infused at 10 μg/min. Five minutes later, the blood pressure returned to 105/65 mmHg, and allergic symptoms resolved within 15 min. Postoperative serum tryptase was 18 ng/mL (normal <11.4 ng/mL), with suspected allergen identified as coagulation factors in plasma. The patient was monitored in the recovery room for 2 h with stable vital signs before being transferred back to the ward, with the affected limb maintained in abduction and neutral position. Preoperative Hb was 85 g/L, immediately after the operation Hb was 110 g/L, and one day after the operation Hb was 105 g/L. There were no symptoms related to anemia (such as dizziness or fatigue).

Postoperative period: The patient developed multiple bright red erythematous lesions with pronounced edema on the trunk and extremities, some coalescing into larger plaques, consistent with acute urticaria. Immediate treatment included intravenous dexamethasone (5 mg), intravenous infusion of 0.9% NaCl (250 mL) with vitamin C (10 mL), oral desloratadine (1 tablet daily), and oral clemastine fumarate (10 mL twice daily). The cutaneous lesions resolved within 30 min of intervention.

The postoperative care emphasized “multidisciplinary perioperative management”, integrating hormonal regulation, complication management, functional rehabilitation, nutritional support, and infection control. For “hormonal modulation”, a “basal dose + stress supplementation” protocol was maintained. Hydrocortisone infusion was tapered gradually based on vital signs and serial monitoring of serum cortisol and thyroid function (FT3, FT4, TSH), transitioning the patient to preoperative oral prednisone (5 mg AM, 2.5 mg PM) and levothyroxine (25 μg/day). serum cortisol levels showed a gradual upward trend: 102 nmol/L (POD1) → 115 nmol/L (POD2) → 125 nmol/L (POD4) → 135 nmol/L (POD8), which met the taper triggers (cortisol > 100–130 nmol/L) and confirmed the safety of dose reduction. TSH levels remained stable at 0.32–0.35 uIU/mL, with no signs of adrenal insufficiency or hypothyroidism exacerbation.

**Figure 4:**
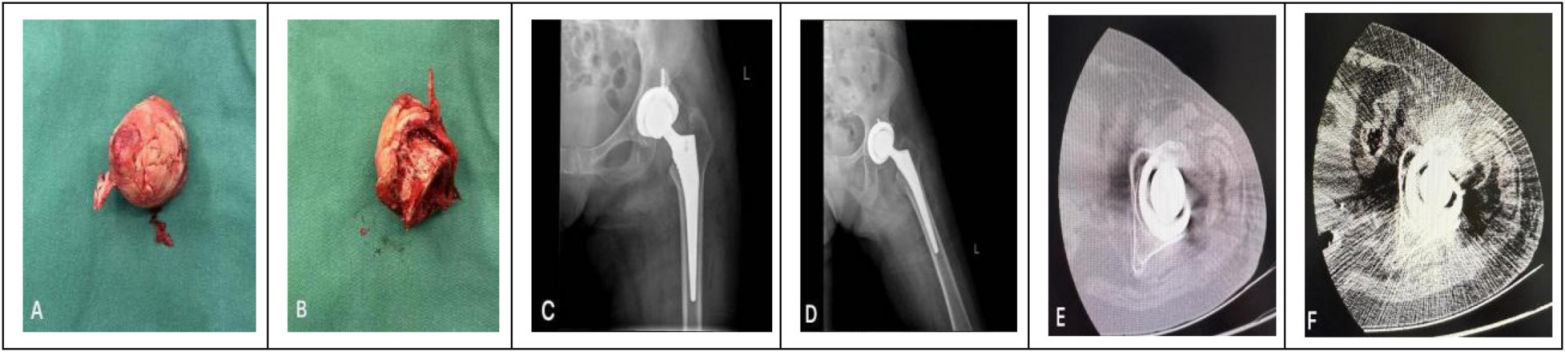
Intraoperative and early postoperative images. **(A,B)** Necrotic femoral head removed during surgery (arrow indicates collapse and deformity); **(C,D)** left hip anteroposterior x-ray at 4 days postoperatively (arrow indicates well-aligned acetabular cup and femoral stem, no loosening). **(E,F)** Postoperative 3-month imaging of the left hip.

On postoperative day 4, left hip radiographs (AP/lateral views) confirmed proper prosthetic positioning without loosening or dislocation (Figure 4). Hepatic function was monitored daily to adjust hepatoprotective therapy, while surgical incisions and lower extremities were examined for signs of infection, prosthesis instability, or deep vein thrombosis. At 3 months postoperatively, hip CT showed bone ingrowth into the porous surface of the acetabular cup, and no periprosthetic radiolucent lines—these findings confirmed good bone-prosthesis integration (Figure 4).

## 4 Follow-up

The patient was followed up for 3 months.Postoperative rehabilitation adopted a phased progression: From POD1-2, ankle pump exercises and passive hip flexion were performed to prevent deep vein thrombosis; from POD3, walker-assisted ambulation (10 min/session, twice daily) was initiated, with gradual increase in duration to 30 min/session at 1 month postoperatively. At 3 months, the patient completed unassisted walking and stair climbing training, which was consistent with the rehabilitation goals of restoring independent daily activities.The hip joint CT scan showed that the two acetabular screws (with a diameter of 4 mm) were not loose or broken, and there was no bone resorption around the screws. Preoperatively, she suffered from severe hip pain and required two crutches for ambulation, with a maximum walking distance of approximately 500 m. Her Harris Hip Score was 19, Visual Analogue Scale (VAS) pain score was 8, and WOMAC Score was 92. One week postoperatively, the patient reported that left hip pain was significantly alleviated; she could move around indoors independently with a walker and dress herself without family assistance. At this time, her Harris Hip Score was 52, VAS score was 3, and WOMAC Score was 58. One month after discharge (one month postoperatively), the patient stated that she experienced mild soreness in the left hip after walking 1 kilometer, but could walk without a cane and was able to help with grocery shopping for her family. Her Harris Hip Score was 82, VAS score was 1, and WOMAC Score was 21. Three months postoperatively, the patient reported no hip pain at all and could accompany her grandson to the park for 1 h. Her Harris Hip Score was 92, VAS score was 0, and WOMAC Score was 6.

## 5 Discussion

This case report documents the comprehensive treatment of a patient with Sheehan syndrome-induced osteonecrosis of the femoral head (ONFH) who underwent total hip arthroplasty (THA) within a multidisciplinary team (MDT) approach. The patient had complex underlying comorbidities, including endocrine dysfunction and malnutrition, attributable to long-term glucocorticoid replacement therapy (11). Long-term glucocorticoid administration can damage vascular endothelial cells, promote microthrombus formation, and enhance bone marrow adipocyte proliferation, ultimately leading to impaired blood supply to the femoral head and diminished bone repair capacity (12). THA effectively restored hip joint function by replacing the necrotic femoral head and compromised acetabulum, resolving joint collapse and severe functional impairment. The MDT framework, incorporating a “basal dose plus stress supplementation” protocol from endocrinology specialists, prevented adrenal crises triggered by surgical trauma and optimized perioperative safety. This outcome aligns with established principles for managing glucocorticoid-induced ONFH. Studies emphasize that THA represents the definitive therapeutic option for late-stage glucocorticoid-induced ONFH (e.g., ARCO stage IIIb or higher), and MDT integration can reduce perioperative complication rates to levels approaching those in standard patients (11). In this case, the MDT strategy also included preoperative nutritional supplementation, intraoperative prosthetic optimization for enhanced fixation, and postoperative infection prevention measures, substantially improving quality of life (transitioning the patient from non-ambulatory status to independent mobility with walker assistance) and increasing treatment adherence (11).

To further contextualize the present case, we summarized previously published reports of Sheehan syndrome or hypopituitarism complicated by osteonecrosis of the femoral head treated with total hip arthroplasty (Table 4). Most reported patients were middle-aged or elderly women receiving long-term glucocorticoid replacement therapy, highlighting steroid exposure as a shared etiological factor for ONFH. Surgical outcomes were generally favorable, with significant pain relief and functional improvement, and serious complications were uncommon. Compared with prior reports, the present case is distinguished by the detailed description of structured multidisciplinary perioperative management, including endocrine optimization, anesthetic planning, nutritional support, and postoperative rehabilitation, which may have contributed to the favorable clinical outcome despite the patient's complex endocrine and metabolic status.

**Table 4:** Published cases of Sheehan syndrome or hypopituitarism complicated by osteonecrosis of the femoral head treated with total hip arthroplasty.

Author (year)	Country	Age/sex	Etiology of hypopituitarism	Cause of ONFH	Surgical intervention	Outcome	Complications
Karaca et al. (2016) (13)	Turkey	58/F	Sheehan syndrome	Long-term glucocorticoid replacement	Unilateral THA	Improved hip function	None reported
Park et al. (2020) (14)	Korea	55/F	Hypopituitarism (non-tumoral)	Steroid-induced ONFH	THA	Pain relief, restored ambulation	None reported
Wang et al. (2019) (12)	China	49/M	Secondary hypopituitarism	Glucocorticoid-associated ONFH	Hip-preserving surgery → THA	Functional improvement	Not specified
Agarwal et al. (2023) (15)	USA	62/F	Multiple pituitary hormone deficiency	Long-term hormone replacement	THA	Significant functional recovery	None reported
Present case	China	63/F	Sheehan syndrome	Long-term glucocorticoid replacement	Unilateral THA with MDT management	Marked pain relief and functional recovery	Transient transfusion-related allergic reaction

In this case, the THA integrated with an MDT approach exhibited distinct advantages. Firstly, the MDT framework facilitated multidisciplinary coordination, enabling seamless integration of endocrine regulation, nutritional support, and orthopedic surgery. This established a closed-loop management system that effectively mitigated risks associated with hormonal imbalances and nutritional deficiencies, which might be overlooked under unidisciplinary care, thereby optimizing patient safety protocols. Secondly, orthopedic procedures were tailored for patients with reduced bone mass, including precise osteotomies and adjunctive screw fixation to enhance prosthesis stability, which minimized complications and improved outcomes (2). The therapeutic regimen demonstrated favorable safety; adverse events were promptly controlled using predefined emergency measures, resulting in no severe consequences. Postoperatively, patient hip function progressively recovered, indicative of the model's efficacy in enhancing quality of life (12). However, the study has two notable limitations that may limit its generalizability. Primarily, functional recovery and complications (e.g., dislocation, infection, or neurovascular injuries) were assessed solely through qualitative descriptions, lacking objective measurements across multiple timepoints (12). Secondly, there was insufficient detail in tracking the long-term optimization of hormone replacement therapy; perioperative hormone level fluctuations and dose adjustments were not recorded, and bone mineral density data—critical for evaluating osteoporosis-related periprosthetic fracture risks—were absent, while the efficacy of such interventions remains unstandardized and prone to adverse events.

Future research should prioritize the following directions: First, inclusion of patients with Sheehan syndrome at varying stages of bone involvement or ONFH progression and differing durations of hormone replacement therapy to validate the long-term safety and efficacy of integrating total hip arthroplasty (THA) with a multidisciplinary team (MDT) approach for comprehensive management, especially in cases with osteoporosis or avascular necrosis (14, 15). Second, refinement of perioperative glucocorticoid regulation protocols, such as establishing optimal hydrocortisone infusion dosage, precise timing during surgical stress, and systematic postoperative tapering schedules to address adrenal insufficiency in hypopituitarism (13). Third, exploration of synergistic applications of bone-preserving agents, including bisphosphonates, in combination with existing therapies to enhance bone mineral density and reduce complication risks, leveraging the osteoprotective effects observed in osteoporosis models. Fourth, development of a dedicated assessment system incorporating endocrine markers, bone mineral density metrics, nutritional status, and other physiological parameters to enable preoperative risk stratification and individualized postoperative follow-up, as advanced imaging and multi-parameter approaches are critical for monitoring treatment outcomes (15).

## 6 Conclusion

In summary, the multidisciplinary team (MDT) approach integrated with total hip arthroplasty (THA) holds pivotal value in managing steroid-induced osteonecrosis of the femoral head (ONFH) secondary to Sheehan syndrome, a condition characterized by hypopituitarism due to postpartum pituitary necrosis (13). This strategy leverages advantages such as coordinated multidisciplinary collaboration, precise perioperative control, and comprehensive care protocols, providing safe and effective treatment for patients with high-risk conditions including steroid imbalances and malnutrition.

## Data Availability

The original contributions presented in the study are included in the article/Supplementary Material, further inquiries can be directed to the corresponding author.
